# Combined and Isolated Effects of Acute Exercise and Brain Stimulation on Executive Function in Healthy Young Adults

**DOI:** 10.3390/jcm9051410

**Published:** 2020-05-10

**Authors:** Erika K. Hussey, Eduardo B. Fontes, Nathan Ward, Daniel R. Westfall, Shih-Chun Kao, Arthur F. Kramer, Charles H. Hillman

**Affiliations:** 1 Soldier Performance Optimization Directorate, US Army Combat Capabilities Development Command Soldier Center, Natick, MA 01760, USA; 2 Center for Applied Brain and Cognitive Sciences, Tufts University, Medford, MA 02155, USA; eduardobfontes@gmail.com (E.B.F.); Nathan.Ward@tufts.edu (N.W.); 3 Department of Physical Education, Federal University of Rio Grande do Norte, Natal CEP 59078-970, RN, Brazil; 4 Center for Cognitive and Brain Health, Northeastern University, Boston, MA 02115, USA; westfall.d@husky.neu.edu (D.R.W.); a.kramer@northeastern.edu (A.F.K.); c.hillman@northeastern.edu (C.H.H.); 5 Department of Health and Kinesiology, Purdue University, West Lafayette, IN 47907, USA; shihchunkao@gmail.com; 6 Beckman Institute, University of Illinois at Urbana-Champaign, Champaign, IL 61801, USA

**Keywords:** aerobic exercise, tDCS, inhibition, working memory, attention

## Abstract

Acute cognitive enhancement has been sought by healthy young individuals to improve academic and professional performance. Among several methods, physical exercise interventions and transcranial direct current brain stimulation (tDCS) have shown promise in impacting executive functions. Here, we observed a set of new findings about the causal effect of acute aerobic exercise and tDCS across three facets of executive function: Inhibition (as measured by a flanker task) was selectively impacted by acute aerobic exercise but not tDCS, whereas working memory (as measured by an n-back task) was impacted by both acute aerobic exercise and tDCS, with effects emerging on distinct processing components for each manipulation. Sustained attention (as measured by the Mackworth clock task), on the other hand, was not impacted by acute aerobic exercise or tDCS. Interestingly, no effects of combining acute aerobic exercise and tDCS emerged. We argue that understanding the unique and combined contributions of these cognitive enhancement techniques can not only contribute to a deeper mechanistic explanation in healthy individuals but also inform future research with clinical and aging populations.

## 1. Introduction

The rising demand to temporarily increase cognitive performance has generated a surge in randomized control trials and neurotechnology development. Two low-cost, non-invasive cognitive enhancement approaches are physical exercise interventions and transcranial direct current brain stimulation (tDCS). This is, in part, because several reports suggest that a single bout of exercise [1,2] or tDCS [3,4] can lead to improvements in executive function (EF) or the general-purpose mental skills required to regulate attentional resources. One proposed mechanistic explanation for such effects involves modulation of the cellular, chemical, and network properties of the prefrontal cortex (PFC), which putatively supports EF [5,6]. Specifically, with the right parameters in place, exercise [7] and tDCS [8] facilitate PFC resources, which are then realized as performance boosts on outcome measures and tasks that require EF. Despite this framework and its clear predictions, results of many exercise and tDCS studies still remain varied and often report small-to-moderate effect sizes [1]. Thus, new studies have turned to multimodal approaches by combining methods to test and promote effectiveness [9,10,11,12]. Many of these designs, however, adopt multi-session intervention timelines, leaving a gap in the literature on *acute* multimodal approaches [13]. The current study investigates the isolated and combined impact of acute physical exercise and single-session tDCS on EF among a sample of young, healthy adults. Cognitive performance was assessed using a battery of tasks that represent three well-established EF processing components: inhibition, working memory, and sustained attention [14,15]. Indeed, the extant literature points to positive effects of exercise and tDCS for each of these processes, yet no work to date has evaluated all three processes within the same individuals within a design that parametrically introduces exercise and tDCS.

### 1.1. Acute Exercise and Executive Function

Acute exercise—a single session of moderate- to high-intensity running, walking, or cycling—reveals promising results across a range of EF processes [1]. Indeed, a meta-analysis indicated that short bouts of aerobic exercise have small but positive effects on behavioral performance of EF tasks performed following exercise [16,17]. Consistent with these findings, a recent review suggests that exercise-induced improvements on EF correspond to increased recruitment of PFC regions and increased electrical brain activity in response to a stimulus [18]. Several mechanisms have been suggested to explain such benefits, including changes in neurophysiological and neurochemicals parameters induced by exercise [18], as well as increased cerebral blood blow and oxygenation accompanied by higher activation in PFC [19].

Of the many EF processes explored to date, inhibition is the most investigated among healthy, young individuals completing short bouts of exercise. For instance, Kao and colleagues (2017) reported that high-intensity interval training on a treadmill (relative to a seated control, rest condition, and a moderate-intensity continuous exercise) induced faster response times on a modified flanker task performed after exercise, as well as smaller amplitudes and shorter latencies of the P3 (an event-related potential within EEG experiments that indexes attention allocation), while moderate-intensity continuous exercise yielded large P3 amplitudes relative to rest and high-intensity interval training conditions [20]. A series of complementary studies using moderate-intensity treadmill paradigms compared to rested control conditions report improved flanker performance after exercise, including increases in P3 amplitude [21], or faster response times and increased P3 amplitudes [11]. This profile of findings provides evidence that inhibition is sensitive to acute bouts of exercise among healthy young adults.

Working memory is another component process of EF that has been shown to benefit from acute bouts of exercise. A meta-analysis revealed that acute exercise leads to moderate increases in working memory (as measured via the n-back task and span tasks), and like the inhibition measures reviewed above, effects in working memory are bolstered by changes in the PFC [22]. For instance, in one study, the authors examined PFC activation using fMRI after 20 min of intense cycling, and they found that relative to a seated control condition, the exercise group had elevated PFC activation during an n-back task [19]. Although these results were not accompanied by behavioral improvements on the n-back task, other studies have provided converging evidence of such behavioral changes. One such report revealed faster response times [23] and increased accuracy on versions of an n-back task completed after moderate bouts of cycling relative to seated control conditions [24]. Together, there is rising evidence suggesting that acute exercise experienced at moderate intensities has positive effects on working memory.

In addition to inhibition and working memory, there also exist a handful of investigations of the effects of acute exercise on sustained attention and vigilance. For instance, relative to visual search performed at rest, target detection response times were faster for individuals performing short 10-min bouts of moderate-intensity cycling [25]. During extended exercise conditions (i.e., for 2 h; [26]) or after a significant delay following high-intensity exercise [27], participants showed no target detection improvements on visual search tasks relative to resting control conditions. This indicates that sustained attention may only be impacted by acute exercise on short time scales, which is what we seek to test in the present study.

Taken together, there is a wealth of findings indicating that a short bout of physical activity can have a positive influence on EF, including but not limited to performance on inhibition, working memory, and sustained attention tasks. Surprisingly little work has examined the effect of acute exercise on multiple EF measures within the same individuals. Weng and colleagues (2015) present one exception, where they tested the effects of 30 min of moderate-intensity cycling on working memory and inhibition in a within-subjects design [24]. The present study seeks to extend this work in several ways. First, we included a suite of EF tasks in a procedure to investigate 20 min of exercise on a treadmill (instead of 30 min on an ergometer). Second, in addition to the n-back task and flanker task, we included a sustained attention measure (i.e., the Mackworth clock task) in an effort to understand how physical activity within the same participants influences performance on a broader collection of EF components. To our knowledge, this particular vigilance task has not been used as an outcome measure in any acute exercise studies; thus, we present the first data on Mackworth clock task performance following physical activity. Lastly, our study compared the effects of acute exercise, single-session tDCS, and combined exercise/tDCS to explore the EF boundary conditions of each enhancement technique.

### 1.2. Single-Session tDCS and Executive Function

Transcranial direct current stimulation is a non-invasive brain stimulation tool that has generated recent interest as a means to improve cognition by modulating the activity of supporting brain regions [28,29]. The leading mechanistic explanation of tDCS-mediated changes in cognitive performance is that the electric field—produced by a low-voltage current that is applied through electrodes placed on the surface of the head—alters the resting membrane potential of neurons in targeted brain regions, like the lateral prefrontal cortex for EF [8,30,31]. Although this change occurs below neurons’ firing thresholds, it provides enough change to elevate (as in the case of anodal simulation) or lower (as in the case of cathodal stimulation) the likelihood of spontaneous action potentials. Numerous studies have demonstrated improvements in behavioral performance on EF tasks during or following tDCS application over PFC regions, with particular success among clinical populations [32]. Using tDCS as a tool to improve EF in healthy adults remains somewhat controversial, however, with a majority of positive effects emerging under highly selective task conditions and often with small effect sizes.

A growing literature indicates that performance on inhibition measures can change with tDCS application over lateral prefrontal regions that serve as primary nodes of EF networks. For instance, anodal tDCS over dorsal lateral PFC elicits boosts in speed and accuracy on the flanker task. For example, Gbadeyan and colleagues (2016) reported boosts in trial-by-trial response times following 20 min of 1 mA of anodal tDCS over left and right dorsolateral prefrontal cortex (DLPFC) relative to sham control [33]. Similarly, smaller Flanker interference effects emerge for 20 min with 1 mA, 1.5 mA, or 2 mA of anodal stimulation over left DLPFC relative to sham control [34]. In a separate study using high-definition tDCS (via a 4 × 1 ring of stimulation), 2 mA of anodal tDCS over right DLPFC led to more accurate flanker performance alongside slower overall response times [35]. Taken together, inhibition is affected by anodal tDCS over lateral PFC for tasks that all share the need to inhibit prepotent information and responses.

Working memory outcomes have also been at the center of extensive recent study when it comes to examining the impact of tDCS on cognitive processing. The n-back task has generated the most results to date, which lack consensus across the literature. In one camp, a series of meta-analyses indicate that tDCS over lateral prefrontal regions improves response time on n-back with little to no change on accuracy [3,4,36]. This is in contrast to a meta-analysis by Horvath et al. (2015) that reported that tDCS induced only a small effect on accuracy and no effects on response time on n-back performance [37]. Altogether, these findings point to a mixed yet promising avenue of investigation into the effects of lateral prefrontal tDCS on working memory [38].

One final EF process that has been well-represented within the tDCS literature is sustained attention or vigilance [39,40]. Specifically, tDCS over left DLPFC is associated with improved performance on the Mackworth clock task, where individuals must successfully and quickly detect infrequent targets under monotonous conditions [41,42]. These sustained attention findings, alongside the collection of working memory and inhibition effects reviewed above, suggest that administering anodal tDCS over lateral PFC can elicit a positive change in performance (whether in terms of minimizing errors or speeding response times). However, little work has explored the impacts of tDCS across these various measures within the same individuals. The extant reports that include more than one EF process are in the context of multi-modal training (see [12]), test the impact in special populations (see [43] for an investigation with older adults), or utilize a battery of tasks not specific to classic EF tasks (see [44,45] for instances incorporating language measures).

### 1.3. Current Study Design and Predictions

The current study seeks to address several knowledge gaps by providing a glimpse into the relative and combined impact of two well-established cognitive enhancement techniques (tDCS and physical exercise) on a battery of executive function tasks that have otherwise not been studied in combination within the same sample of individuals: inhibition, working memory, and sustained attention. In addition, our study contributes to and extends existing research in several ways. The most obvious extension is to test the relative and combined impact of the two techniques in a fully crossed design (i.e., exercise vs. tDCS vs. both vs. rested control). Furthermore, we test this effect on three different executive function components in a within-subjects design. Finally, we explore this research question in a cohort of young, neurotypical adults for whom we include a comprehensive set of accuracy and response latency metrics.

We predicted that active exercise would lead to better performance than rest and that active tDCS would lead to better performance than sham tDCS. With regard to the combined effects of tDCS and exercise, we predicted that both methods presented in combination would lead to superior performance beyond either method in isolation if both techniques impact common neural mechanisms supporting EF.

## 2. Materials and Methods

### 2.1. Study Design

**Figure 1:**
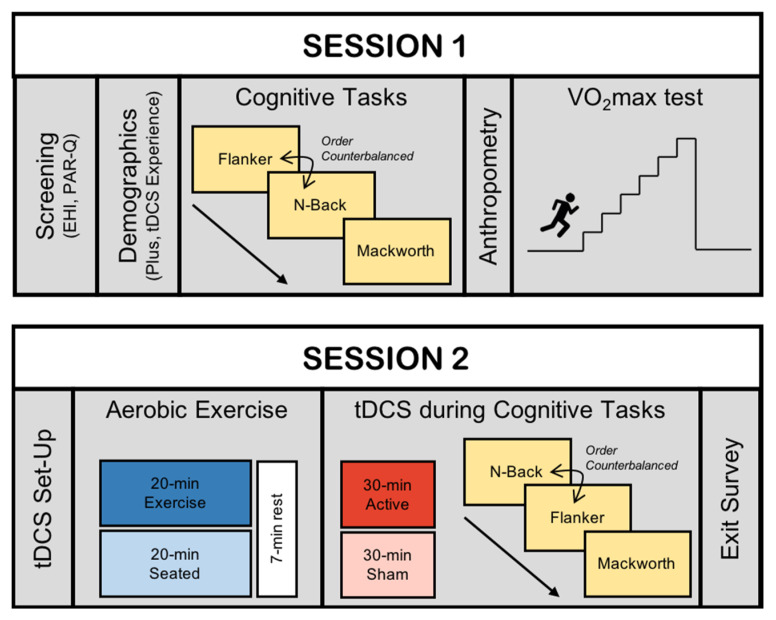
Overview of the timeline of activities at each experimental session. Session 1 activities appear in the top panel and Session 2 activities appear in the bottom panel. tDCS = transcranial direct current stimulation.

Participants were enrolled in two separate testing sessions separated by an average of 5.5 days (range: 1 day to 31 days, with 89% of participants completing both sessions with a week or less). In the first session, they completed three cognitive tasks to obtain baseline assessments of inhibition, working memory, and sustained attention. They then completed a maximal incremental cardiorespiratory fitness test to determine individualized cardiorespiratory fitness for the exercise task in Session 2. In the second testing session, participants returned and were randomly assigned to run or remain seated and to receive active or sham tDCS, creating a 2 × 2 design of four between-subjects testing conditions. Specifically, upon arriving at Session 2, half of the participants were randomly selected to complete a 20-min aerobic exercise bout on a treadmill or to remain seated for 20 min. Following this period of time, half of the participants in each group were randomly assigned to receive either active tDCS or sham tDCS as they completed the same cognitive tasks performed in Session 1. A summary image of the activities in each session is depicted in Figure 1.

### 2.2. Participants

One hundred seven young neurotypical adults from a college community were recruited to participate in the study for payment. All subjects gave their informed consent for inclusion before they participated in the study. The study was conducted in accordance with the Declaration of Helsinki, and the protocol was approved by the University of Illinois, Urbana-Champaign Institutional Review Board (IRB Protocol Number 15866). Data from 11 participants were discarded due to withdrawal between Sessions 1 and 2 (*n* = 4), ineligibility detected through Session 1 screening questionnaires (*n* = 1), and delivery of less than 2 mA of tDCS current, if requested by the participant (*n* = 6), resulting in a final sample size of 96 total participants (24 in each condition). Demographic information of the final sample appears in Table 1.

### 2.3. Session 1

#### 2.3.1. Screening Procedures

Participants were screened for the following criteria based on self-report: at least 18 years of age; right-handed; fluent in English; normal or corrected-to-normal vision; not pregnant or planning to become pregnant in the next 6 months; no metallic implants above the neck; no implanted internal or external electrical stimulation devices; no neurological conditions that may limit the ability to complete cognitive tasks; no blood pressure medications, herbal dietary supplements, performance supplements, or any other substance that could affect cardiovascular response with exercise; no medications or supplements designed to increase alertness or attention; no non-removable metal or tattoos containing metallic ink on the right bicep; and no injections into the right bicep within the last 6 months. Additionally, at the beginning of Session 1, participants were screened again for the same exclusionary criteria. Handedness was assessed with the Edinburgh Handedness Inventory [46] where left-handed individuals were identified and excluded from the experiment. This is because tDCS effects have been shown to be influenced by hemispheric dominance and handedness [47]. Based on their responses on the Physical Activity Readiness Questionnaire (PAR-Q) [48], individuals with any health issues that may have prevented them from being able to participate in strenuous exercise were excluded from participation. The remaining screening criteria above were verified a second time in person.

#### 2.3.2. Demographics

Participants meeting the eligibility requirements then completed a general questionnaire to assess demographic information including self-reported age, gender, and language use. In addition, they were asked to report their previous experience with and expectations about the efficacy of neurostimulation.

#### 2.3.3. Baseline Cognitive Task Assessment

Participants performed three cognitive tasks to establish baseline performance levels prior to exercising or receiving brain stimulation. The tasks tapped several EFs including inhibition (flanker task), working memory (n-back task), and sustained attention (Mackworth clock task). The order of the flanker and n-back tasks were counterbalanced with the Mackworth clock task always occurring last. Detailed descriptions of each task appear below in the Session 2 section.

#### 2.3.4. Cardiorespiratory Fitness Assessment

Anthropometric assessments of height and weight were taken prior to the fitness assessment (see Table 1). Maximal oxygen consumption (VO_2_max) was measured using a computerized indirect calorimetry system (ParvoMedics True Max 2400) with averages for oxygen uptake and respiratory exchange ratio (RER) assessed every 20 s. A modified Balke protocol was employed using a motor-driven treadmill (Life Fitness 93T) at a constant speed with 2.0% increases in grade every two minutes until volitional exhaustion was reached. A Polar heart rate (HR) monitor (Model A1, Polar Electro, Finland) measured HR throughout the test, and ratings of perceived exertion (RPE) [49] were assessed every two minutes during the test. Relative peak oxygen consumption was expressed in mL/kg/min and was evidenced by the participant achieving two of the following four criteria: (1) a plateau in oxygen consumption corresponding to an increase of less than 2 mL/kg/min despite an increase in workload; (2) HR within 10 beats per min (bpm) of age-predicted maximum (i.e., 220-age; HR_max_); (3) RER greater than 1.10; or (4) an RPE greater than or equal to 17. Group averages of maximal oxygen consumption, maximum HR, and baseline HR during the fitness assessment can all be found in Table 1.

### 2.4. Session 2

#### 2.4.1. Aerobic Exercise Manipulation

In the second testing session, participants were randomly assigned to either 20 min of aerobic exercise on a treadmill (fast walking with modulated incline) or sitting. For the Exercise condition, participants exercised on a treadmill for 20 min. A 2-min warmup period at 3 mph was followed by 16 min of exercise at an intensity of 60–70% of the individual HR_max_ reached in the VO_2_max test during the first session, which was achieved by adjusting the treadmill incline at the beginning of every minute. Subsequently, a 2-min resting period was given at a speed of 3 mph. For the Seated condition, individuals were seated on a chair positioned on the treadmill for the same amount of time as the aerobic exercise condition. During both conditions, a heart monitor (EZ 600/200, Polar, Finland) was used to collect HR data and monitor exercise intensity. After exercising on the treadmill, participants were seated in a chair in the testing room to rest for 7 min to allow HR to return to baseline before beginning the tDCS administration and cognitive tasks. The resting condition was matched to include an additional 7-min buffer as well.

#### 2.4.2. tDCS Manipulation

Brain stimulation montages were prepared prior to exercising or sitting on the treadmill to allow for a smooth transition from the physical activity portion of the session to the seated tDCS portion. Additionally, the extra time spent wearing the electrode montages enabled optimal contact quality to be achieved between the electrodes and the scalp [50]. Each participant’s scalp was measured to identify the left dorsolateral prefrontal cortex (DLPFC) or F3 based on the international 10–20 system [51]. Anodal stimulation was delivered uniformly across five electrodes arranged in a circular array (Mind Research Network) [52] that was centrally placed over the left DLPFC and served as the anode. A comparable 5-electrode array served as the cathode and was placed on the right bicep. Prior to electrode placement, both sites were cleaned with alcohol pads and treated with highly conductive electrode gel. Arrays were secured with bandages. Supplies and montages were carefully inspected, and contact quality/impedance was assessed prior to and following the bout of running or sitting.

Following the physical activity portion of the session, participants moved to a desk with a computer. Both electrode arrays were connected to a Soterix Medical 1 × 1 Transcranial Direct Current Low-Intensity Stimulator through which the current was administered. Specifically, a 2 mA tDCS current ramped up over the course of 30 s, and either remained at this intensity for a 30-min period before ramping down (Active tDCS) or immediately ramped-down within 30 s (Sham tDCS). At the end of the 30-min administration period, the current ramped down in the active condition and repeated the ramp-up/ramp-down sequence in the sham condition [53]. Participants were instructed to complete 3 cognitive assessments about 3 min after the ramp-up period. This resulted in online tDCS administration for the first 2 cognitive tasks (inhibition and working memory) and offline stimulation for the final task (sustained attention).

#### 2.4.3. Cognitive Assessments

Participants completed the 3 cognitive tasks that were baselined in Session 1. The order of the first two tasks (flanker and n-back tasks) were counterbalanced, and the Mackworth clock task always occurred last. We decided to fix the final task due to its length (i.e., approximately 30 min versus the other two tasks, which were each no longer than 15 min) and to maximize the number of tasks being administered with online tDCS.

##### Inhibition (Flanker Task)

A modified flanker task was employed, in which an array of five arrows was presented centrally on a black background of a computer screen using E-Prime 2.0. Participants were instructed to respond to the directionality of the central target arrow using a thumb press on a response pad as quickly and accurately as possible. Congruency was varied by manipulating the directionality of the flanking arrows. Flanking stimuli were either congruent (i.e., all arrows facing in the same direction, < < < < < or > > > > >) or incongruent (i.e., flanking arrows facing the opposite direction, < < > < < or > > < > >). Congruency and directionality were equiprobable and stimuli were presented randomly. Stimuli were presented for 100 ms with a variable inter-stimulus interval of either 900, 1100, or 1300 ms. Participants completed 36 practice trials before completing two blocks of 144 trials, resulting in a task that took no more than 15 min to complete.

##### Working Memory (n-Back Task)

Participants performed an n-back task where they indicated whether a current letter matched or mismatched the letter presented n trials prior. N-level was manipulated to introduce a high demand on working memory [54,55]. Specifically, participants first completed a block of 2-back sequences followed by a block of 4-back sequences. Each block began with 1 practice sequence followed by 5 test sequences. Every sequence contained 6 targets (i.e., n-back matches), 6 interference lures (i.e., repeated but mismatched items in non-n-back positions), and n + 10 fillers (i.e., mismatches that were not repeated items). Thus, the 2-back sequences each had 22 trials, while the 4-back sequences contained 24 trials, resulting in a 15-min task. The purpose of this design feature was to ensure an equal number of eligible target items on all sequences, regardless of n-level (see [56]). The letters (b, c, d, f, h, j, k, l, m, p, q, r, s, t, v, x) were presented serially for 500 ms with an inter-stimulus interval of 2 s using Paradigm Player software. Trial level and sequence level responses and latencies were output for analysis.

##### Sustained Attention (Mackworth Clock Task)

Participants completed an adapted version of the Mackworth clock task during which they were instructed to watch a light move consistently around positions on a circle at a steady pace and report any skipped positions [41]. The display contained 16 small circles arranged in a larger circle. Each of the 16 circles turned red one at a time each for 525 ms moving in a predictable clockwise sequence. Participants were asked to indicate via speeded button press whenever a circle position was skipped (i.e., when the red light appeared to move twice the distance). There were 12 targets among 3442 total stimuli, resulting in a 30 min task. Adopting McIntire et al. (2014)’s scoring, when a target was correctly detected within 8 s, the response was coded as a hit. If it was detected after 8 s, it was coded as a false alarm. If it was not detected, it was coded as a miss. Response type was recorded for analysis.

#### 2.4.4. Exit Survey

After completing the entire experiment, we then asked participants to report on their experiences with the neurostimulation, including guessing which condition they were assigned to (active vs. sham)s and indicating ratings of cutaneous sensation on the dimensions of distraction, itchiness, pain, heat, and discomfort. The purpose of gathering this information was to determine if there were perceived differences between the sham and active conditions.

### 2.5. Statistical Analysis

We assessed the impact of acute exercise and tDCS on a series of accuracy and speed metrics in three EF tasks. Measures of accuracy included traditional average accuracy/error rates, as well as non-parametric signal detection parameters to evaluate target/non-target sensitivity and response bias [57]. Measures of speed included median response latencies and response latency standard deviation. We evaluated reaction time standard deviations because variability in latency corresponds to performance stability, which may otherwise be masked with measures of central tendency [58,59]. We additionally report ex-Gaussian parameters of central tendency, variance, and skew for correct response times, given that these models provide a more accurate characterization of speed response time distributions [60,61] (for implementations in aerobic exercise interventions, see [62,63]; for implementations in tDCS designs, see [64,65]).

All metrics collected at Session 2 (when aerobic exercise and/or tDCS were introduced) were corrected for Session 1 performance, or a difference score from Sessions 1 to 2. Difference scores for accuracy metrics were all expected to increase over time, so Session 1 was subtracted from Session 2. Speed metrics were all expected to decrease over time, so Session 2 was subtracted from Session 1.

All statistical analyses were performed using RStudio (Version 1.1.463) and R (Version 3.5.1). Linear mixed-effects models (LMEs) were analyzed using the “lme4” package (Version 1.1.19), and ex-Gaussian simulations were derived using the “retimes” package (Version 0.1.2; for a review on ex-Gaussian approaches, see [66]). For all analyses, a coefficient t-value with an absolute value magnitude greater than 2.00 was considered statistically significant [67]. Note that all analyses were verified with Kenward-Rogers approximations of *p*-values as well (see [68,69]).

#### 2.5.1. Inhibition (Flanker Task)

Flanker metrics were modeled using LMEs. All fixed effects were Helmert contrast coded to enable comparison across levels of each factor and to weigh each group level according to number of observations where these differed across levels [70]. The models included fixed effects of tDCS (Active: 0.5, Sham: −0.5), Aerobic Exercise (Exercise: 0.5, Seated: −0.5), and Congruency (Congruent: 0.5, Incongruent: −0.5), and random intercepts of Subjects (*N* = 96). Flanker accuracy was evaluated using Average Accuracy, and speed was evaluated using Median Correct Response Time, Correct Response Time Standard Deviation, and ex-Gaussian parameters of central tendency (μ), variance (σ), and skew (τ). For all speed metrics, incorrect items were removed, or 7.68% of the data. Of the remaining correct trials, trials with RT < 200 ms were also excluded, or 0.078% of the correct trials that survived the accuracy filter.

#### 2.5.2. Working Memory (n-Back Task)

n-back metrics were also modeled with LMEs. The models included fixed effects of tDCS (Active: 0.5, Sham: −0.5), Aerobic Exercise (Exercise: 0.5, Seated: −0.5), and N-Level (2-Back: 0.5, 4-Back: −0.5) and random intercepts of Subjects (*N* = 96) and Sequences (*N* = 5). We additionally evaluated performance across sequences (or averaged over 22 items in 2-back sequences, and 24 items in 4-back sequences). All participants completed 5 sequences at each n-level; thus, this approach yielded 5 values per n-level per subject (or 960 total values) for each model. n-back accuracy was evaluated using Average Accuracy and non-parametric signal detection parameters of target/non-target discriminability (A’) and response bias (Grier’s β; [44]). n-back speed was evaluated using Median Correct Response Time, Correct Response Time Standard Deviation, and ex-Gaussian parameters of central tendency (μ), variance (σ), and skew (τ). For all speed metrics, incorrect items were removed, or 15.05% of the data. Of the remaining correct trials, trials with RT < 200 ms were also excluded, or 0.54% of the correct trials that survived the accuracy filter.

#### 2.5.3. Sustained Attention (Mackworth Clock Task)

Mackworth performance was assessed with 2 × 2 Analyses of Variance (ANOVA), with tDCS (Active vs. Sham) and Aerobic Exercise (Exercise vs. Seated) as factors. The reason for using ANOVAs instead of LMEs was due to the low dimensionality of the Mackworth data (i.e., fewer observations than what was required to include a random effects structure in the model). Mackworth accuracy was evaluated using Number of Hits, Number of False Alarms, and non-parametric signal detection parameters of target/non-target discriminability (A’) and response bias (Grier’s β). Three participants’ data were removed from analyses who committed more than 50 false alarms in a single task session. A *p*-value less than 0.05 was considered statistically significant.

## 3. Results

### 3.1. tDCS Expectations and Sensations

At the beginning of Session 1, we asked participants to report on their expectations about tDCS to impact cognitive performance on a scale of 1–10, with 1 being “No Expected Changes” and 10 being “Definite Expected Changes”, and found no differences between the four groups (*p* > 0.55). Overall, volunteers were neutral on their expectations for tDCS to change cognition (M = 4.46). At the end of Session 2, we asked participants to report which tDCS condition they thought they were assigned to on a scale of 1–10, with 1 being “Definitely Did Not Receive tDCS” and 10 being “Definitely Received tDCS.” We found no significant effects across group guesses (*p* > 0.12), with ratings suggesting uncertainty about condition assignment (M = 5.05). We also asked subjects to report any sensations associated with tDCS following Session 2, using a questionnaire where they rated how distracting, itchy, painful, hot, and uncomfortable they found tDCS. The groups did not differ on any of these dimensions (*ps* > 0.21) and provided relatively low ratings across the board (see Panel 2 of Table 1), signaling that tDCS sensations were modest. Together with the expectation ratings, these results suggest that the participants overall were unaffected by their experiences and beliefs about tDCS. Thus, any stimulation effects that we observe can be more confidently attributed to changes in neurophysical resources as a result of tDCS application.

### 3.2. Baseline Fitness

During Session 1, all participants completed a VO_2_max test, during which their baseline fitness levels were determined. The middle panel of Table 1 depicts each group’s means and standard deviations on a series of measures on which we ran ANOVAs with Aerobic Exercise (Exercise vs. Seated) and tDCS (Active vs. Sham) as factors to test for baseline differences in the groups prior to random assignment in the second session. We observed a significant effect of tDCS on VO_2_max (*F* = 4.04, *p* = 0.038), such that individuals who were eventually randomly assigned to receive active stimulation had higher VO_2_max levels (50.07 mL/kg/min) than those who were assigned to the sham condition (45.85 mL/kg/min). However, no other measures reflected this difference: Baseline Heart Rate (*ps* > 0.34), Maximum Heart Rate (*ps* > 0.23), Exercise Duration (*ps* > 0.46), Exercise Speed (*ps* > 0.09), and Treadmill Grade (*ps* > 0.50).

### 3.3. Inhibition (Flanker Task)

**Figure 2:**
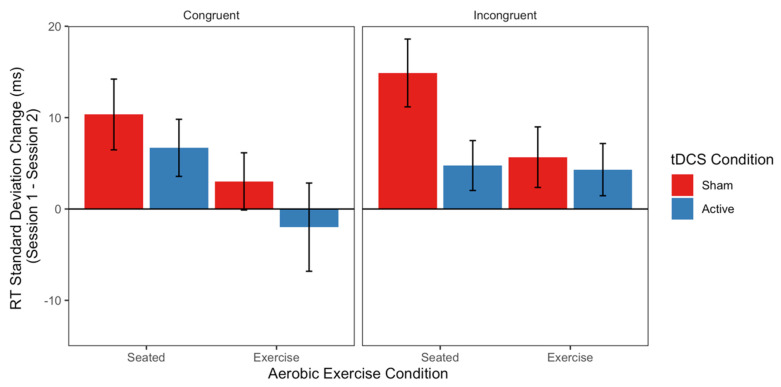
Correct response time standard deviations (in milliseconds, ms) on the flanker task. Bars depict average differences between Sessions 1 and 2 for each Aerobic Exercise condition (Seated vs. Exercise, leftmost and rightmost bars of each panel, respectively) and transcranial direct current brain stimulation (tDCS) condition (Active vs. Sham, in Blue and Red, respectively). Split across two panels is Congruency (Congruent vs. Incongruent, on the left and right, respectively). Error bars reflect standard errors of the mean. RT = Response Time

Linear mixed-effects (LME) models of average accuracy and median correct response time on the flanker task revealed no effects of Aerobic Exercise, tDCS, or Congruency (ts < |1.26|; see top panel of Table 2 for full model output). However, the LME model of response time standard deviation revealed a main effect of Aerobic Exercise (*t* = −2.122), with no other fixed effects or interactions reaching significance (*ts* < |1.66|; see third panel of Table 2 for full model output). Specifically, the Seated groups showed a greater change in RT standard deviation from Session 1 to Session 2 (M change = 9.417 ms) compared to the Exercise groups (M change = 2.752 ms). As can be seen in Figure 2, the leftmost bars in each panel (corresponding to the Seated groups) are markedly more positive than the rightmost bars in each panel (corresponding to the Exercise groups). This suggests that the Seated groups became less variable in their flanker response times from Session 1 to Session 2 than the Exercise groups. Models of all flanker response time ex-Gaussian parameters (μ, σ, and τ) revealed no significant effects of Aerobic Exercise, tDCS, or Congruency (*ts* < |1.99|; see bottom panels of Table 2 for all model outputs).

### 3.4. Working Memory (n-Back Task)

**Figure 3:**
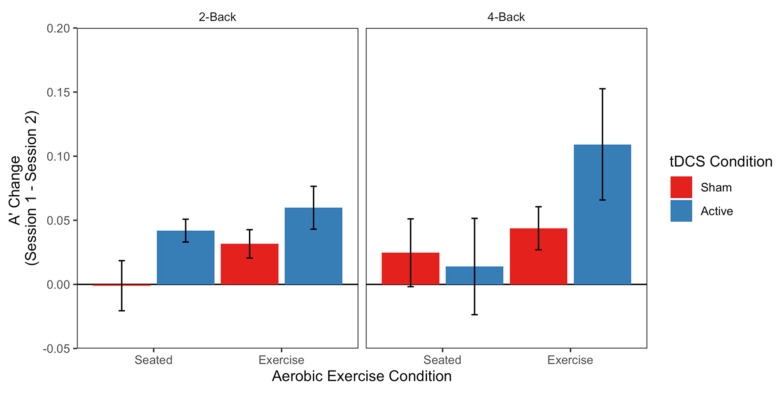
Target/nontarget discriminability (**A’**) on the n-back task. Bars depict average differences between Sessions 1 and 2 for each Aerobic Exercise Condition (Seated vs. Exercise, leftmost and rightmost bars of each panel, respectively) and tDCS Condition (Active vs. Sham, in Blue and Red, respectively). Split across two panels is N-Level (2-Back vs. 4-Back, on the left and right, respectively). Error bars reflect standard errors of the mean.

**Figure 4:**
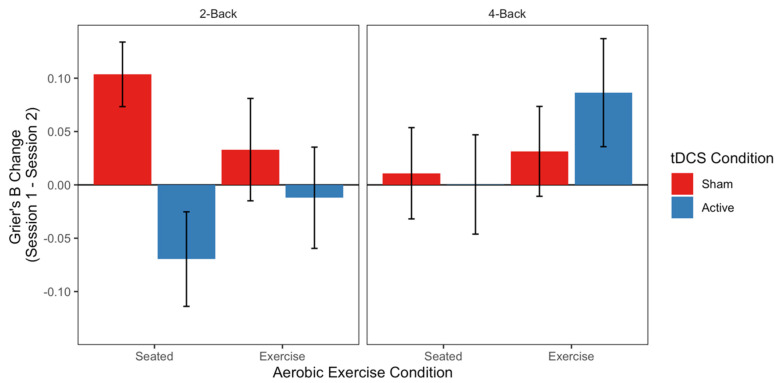
Bias to respond “Target” (Grier’s β) on the n-back task. Bars depict average differences between Sessions 1 and 2 for each Aerobic Exercise Condition (Seated vs. Exercise, leftmost and rightmost bars of each panel, respectively) and tDCS Condition (Active vs. Sham, in Blue and Red, respectively).

LME models of average accuracy on the n-back task revealed no effects of Aerobic Exercise, tDCS, or N-Level (*ts* < |1.87|; see top panel of Table 3 for full model output). However, the LME model of A’ revealed a main effect of Aerobic Exercise (t = 2.266), with no other fixed effects or interactions reaching significance (ts < |1.73|; see second panel of Table 3 full model output). Specifically, the Exercise groups showed overall greater improvements in target/non-target discrimination from Session 1 to Session 2 (M = 0.06 A’ increase) relative to the Seated groups (M = 0.02 A’ increase; see Figure 3). The LME model of Grier’s β revealed an interaction of tDCS and N-Level (*t* = −2.493), with no other significant fixed effects or interactions (*ts* < |1.51|; see third panel of Table 3 full model output). As can be seen in Figure 4, the Sham groups demonstrated more conservative responding (i.e., more likely to respond “non-target”) under low working memory demands (2-Back M = 0.068) compared to situations with higher demands (4-Back M = 0.021). Active groups showed the opposite pattern: they adopted a more conservative response strategy under high working memory demands on 4-Back (M = 0.043) and a less conservative strategy for 2-Back (M = −0.041). Together, the signal detection findings indicate that working memory response decisions are differentially impacted by Aerobic Exercise and tDCS, such that Aerobic Exercise influences item discriminability, while tDCS influences strategic responding. The LME models of all n-back speed measures (correct median response time, correct response time standard deviation, and ex-Gaussian parameters μ, σ, and τ) revealed no significant effects of Aerobic Exercise, tDCS, or N-Level (*ts* < |1.87|; see bottom panels of Table 3 for all model outputs).

### 3.5. Sustained Attention (Mackworth Clock Task)

ANOVAs of number of hits, number of false alarms, A’, and Grier’s β revealed no main effects or interaction of Aerobic Exercise or tDCS (Fs < 1.28, *ps* > 0.26). These null effects suggest that Aerobic Exercise and tDCS each had minimal impact on attention and on-task vigilance.

## 4. Discussion

To summarize, we observed a set of new findings about the causal effect of acute aerobic exercise and tDCS across three facets of executive function: Inhibition (as measured by the flanker task) was selectively impacted by acute aerobic exercise, but not tDCS, whereas working memory (as measured by the n-back task) was differentially impacted by acute aerobic exercise and tDCS, with effects emerging on distinct processing components for each manipulation. Sustained attention (as measured by the Mackworth clock task), on the other hand, was not impacted by acute aerobic exercise or tDCS. Interestingly, no effects of combining acute aerobic exercise and tDCS emerged under the current experimental conditions, suggesting that the influence each of these interventions has on executive function abilities may be partially orthogonal.

**Figure 5:**
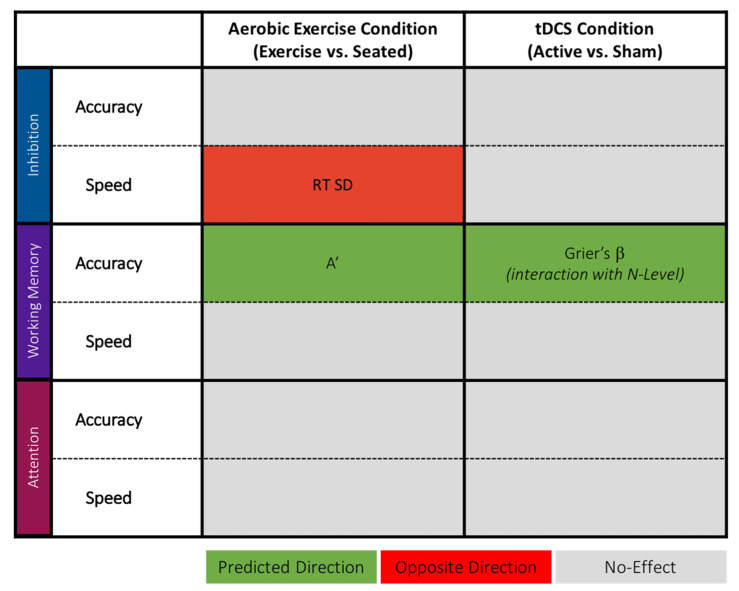
Summary of all significant main effects of Aerobic Exercise and tDCS on inhibition (flanker task), working memory (n-back task), and sustained attention (Mackworth clock task) broken down by measures associated with accuracy (i.e., average accuracy, A’, Grier’s β) and response latency (i.e., correct median response time, correct response time standard deviation (RT SD), ex-Gaussian parameters μ, σ, and τ). Green shaded boxes indicate effects in the predicted direction (see Introduction), red shaded boxes indicate effects in the opposite direction than what was predicted, and gray boxes indicate no effect.

The profile of significant metrics also revealed new evidence as it pertains to the impact of acute aerobic exercise and tDCS on executive function performance. Specifically, as depicted in Figure 5, inhibition performance was only impacted by aerobic exercise, and the effect selectively resided in variability of speed metrics. In contrast, working memory performance had select benefits in only accuracy metrics, with separate effects of Aerobic Exercise (on item sensitivity) and tDCS (on response strategies). Furthermore, sustained attention performance was not significantly affected by Aerobic Exercise or tDCS.

### 4.1. Exercise and tDCS for Improved Inhibition

Inhibition is a widely studied EF within acute exercise and tDCS studies alike, with reports covering effects across different cohorts and populations. Studies in healthy young adults have shown that exercising before performing an inhibition task, like the flanker task, induces low-to-moderate decreases in response time and increases in accuracy [11,20,71]. The results of the present study differ from these studies by showing no effects on RT and accuracy during the inhibition task after exercising while showing effects on RT variability instead. Similar null results have also been shown [72]. More recently, a study showed no acute effects of moderate exercise on behavioral and neural activity during a flanker task performed in an MRI scanner [73]. Therefore, the impact of moderate exercise on traditional parameters (i.e., response time and accuracy) on the flanker task merits further investigation. Instead, we investigated the impact of exercise on non-traditional response time metrics (response time variability) because response time variability in the flanker task has been shown to be related to white matter integrity and functional connectivity [74]. The present study revealed that seated control groups had more consistent response times (i.e., smaller response time variability) relative to the exercise group, which instead showed no substantive change in variability in responding across sessions (Figure 2). This pattern differs from the results of a recent study in a large sample of children, which found that higher cardiorespiratory fitness, which is usually induced by regular sessions of aerobic exercise (i.e., a chronic effect), is associated with lower variability in RT inhibition [63]. It is possible that response time variability patterns on executive function measures vary across the lifespan.

In addition, in the present study, tDCS did not affect any inhibitory control measures that were analyzed. This result contradicts previous studies that showed that tDCS applied over the left DLPFC leads to smaller interference effects [34] and more accurate performance [35] on the flanker task. It is possible that the version of the task that we used was too simple, which might have elicited low variance in performance (i.e., ceiling effects on accuracy and floor effects on RTs); indeed, Karuza and colleagues utilized a version of the flanker task that included go/no-go trials, which would increase the overall difficulty of the paradigm. Finally, there is some evidence linking flanker task performance to right PFC activation [75]. The tDCS montage used in the present study was applied over left PFC, so it is possible that this may explain our null findings. Although some work has explored the impact of tDCS over left versus right PFC on flanker performance [33], these designs utilized different tDCS set-ups and devices that administered the current more focally (i.e., high-definition tDCS) compared to our design. Future work should compare the results of different tDCS protocols and parameters to determine the montage design parameters that give rise to consistent effects on certain EF measures.

### 4.2. Exercise and tDCS for Improved Working Memory

Working memory is another EF routinely tested within cognitive enhancement studies among healthy individuals. Our results show that an acute bout of aerobic exercise improved n-back task accuracy (i.e., target/non-target discriminability; Figure 3). These findings are in line with a metanalytic study that showed that this type of physical activity performed at light-to-moderate intensity in young, healthy individuals leads to consistent increases in working memory performance [22].

Neurostimulation, and tDCS in particular, has also been used to test for improvements in working memory, though the extant findings are not without controversy. Three different meta-analyses suggest that tDCS over DLPFC improves response times but not accuracy across several different working memory tasks [3,4,36]. However, a meta-analysis by Horvath and colleagues (2015) concluded that tDCS has a small impact on n-back task accuracy but not response time [37]. Our results are consistent with this pattern: we found selective benefits of tDCS on n-back in terms of a response criterion shift (a measure derived from accuracy) but did not find any effects on response time measures. One explanation for why we observed effects only for a response criterion measure and no other accuracy measures is because the current n-back task design included a high number of interference lures. Lures are trials that introduce confusability through their heightened familiarity, despite not being target items. Including lures introduces more conservative responding in the form of higher response criteria [56], and this effect is exaggerated when tDCS is applied [44,76].

### 4.3. Exercise and tDCS for Improved Sustained Attention

Neither acute exercise nor neurostimulation had an impact on sustained attention as indicated by performance on the Mackworth clock task. This differs from previous research that has found beneficial effects from these interventions. For example, Barnes and colleagues (2010) found improved attention performance on a dot probe task following a 30-min session of moderate exercise compared to a rest control group [77]; however, others have failed to find any impact of acute exercise on sustained attention performance [78,79]. In a similar vein, Fritz and O’Connor (2016) found that a 20-min session of moderate-intensity exercise reduced fatigue but did not impact vigilance for a group of adults with ADHD [80].

In terms of brain stimulation impacting sustained attention, Nelson and colleagues (2014) stimulated left DLPFC and found improvements in hit rates and false alarm rates on a 40-min simulated air traffic controller task relative to control stimulation, and the effect persisted throughout the duration of the task [81]. Similarly, McIntire et al. (2014) compared the impact of tDCS and caffeine on sustained attention for sleep-deprived participants and found that active tDCS prevented declines in vigilance in both accuracy and response times [41]. In contrast, other studies have found mixed results. For example, tDCS did not affect performance on an attention-to-response task but instead increased mind-wandering [82]. Furthermore, others have argued that PFC stimulation primarily impacts higher-order processes connected with attention rather than simple target detection [40], which presents yet another avenue for future research to explore.

One additional possible explanation for the lack of observed effects is that the participants may have been mentally fatigued by the preceding working memory and inhibition tasks; indeed, the Mackworth task was always the final assessment that was completed.

### 4.4. Combining Exercise and tDCS

Despite an increasing interest in cognitive enhancement techniques for healthy populations, many effects on executive function reported thus far tend to be small, including those presented here. It has recently been proposed that combining techniques through multimodal approaches may promote synergetic mechanisms that increase the likelihood of improved cognitive functioning [10,83]. Much work has investigated simultaneous neurostimulation and physical activity [84], yet only a few reports have evaluated cognitive performance outcomes measures other than EF, like skill acquisition and perceptual learning [85] and perception of effort [86,87,88]. Another study combined high-intensity acute running on a treadmill with transcranial laser stimulation and measured sustained attention on a psychomotor vigilance task (PVT) and working memory using delayed match-to-sample (DMS) working memory task [89]. Although they found faster response times on the PVT and more accurate responding on the DMS task for both groups receiving only exercise and/or only stimulation groups, the combined exercise and stimulation group did not produce an added benefit. Our results are somewhat consistent with this pattern; however, it is worth noting that we used a different form of neurostimulation (direct current versus laser stimulation), and we tested performance on different sustained attention and working memory tasks.

To our knowledge, only one multimodal study involving both exercise and tDCS found improvements in EF processes [12]. Across a 4-month intervention that combined tDCS, physical exercise, and cognitive training, the authors reported improvements in working memory learning rates for the condition that completed all three enhancement techniques compared to a group with only cognitive training and a group with combined cognitive training and exercise. Using an acute intervention, our data showed no marked effects for the combined exercise and tDCS condition relative to the conditions implementing exercise and tDCS in isolation. This could be due to emerging baseline fitness differences in the Active vs. Sham tDCS groups in terms of their VO_2_max levels. It is possible that to gain the positive effects of multimodal cognitive enhancement, an extended period of time with multiple intervention points may be required, as Ward et al.’s longitudinal intervention demonstrates. Thus, although there are several frameworks describing potential shared mechanistic underpinnings of exercise and tDCS [13], understanding when and how both techniques might optimally improve higher-order cognitive processing likely depends on several parameters (including, perhaps, chronic versus acute application).

### 4.5. Future Directions

We acknowledge several limitations in the present work. First, introducing tDCS and exercise in a between-subjects design may not be an ideal comparison. For tDCS, individual differences in neuroanatomy may have profound effects due to the same montage impacting different neural regions. Interestingly, one study has even shown that genetic factors can modulate effects of tDCS on cognitive performance [90]. For exercise, baseline fitness levels of participants vary, which may have incidental effects on cognitive task performance. In the current sample, the active tDCS group was more fit at baseline than the sham group in terms of VO2max levels, and this finding could help to explain why we did not observe significant results distinguishing the combined tDCS and Exercise from the others. However, in our study, the exercise intensity in the second session was prescribed based on individual maximum heart rate, controlling for such individual differences in fitness and physical activity to some extent. Regardless, a future area of study might characterize the impact of baseline fitness as a mediator of responsiveness to combined aerobic activity and tDCS interventions for cognitive performance. This is especially pertinent given the findings of two recent studies which concluded that low fitness negatively impacts executive function, as evidenced by fitter subjects outperforming their less fit counterparts on a modified Stroop task [91,92]. Alternatively, a repeated-measures cross-over design would neutralize the impact of these individual differences that naturally emerge in a between-subjects design by having each participant serve as their own control. Additionally, there are a host of state-based factors (e.g., quality of sleep the night before, time of day for testing, participant stress level) that likely varied across the two experimental sessions; any number of these could have impacted cognitive performance across the sessions regardless of whether a cross-over or between-subjects design is implemented.

Second, there are several testing parameters that may be driving our findings that we did not explicitly test within the current study. For tDCS, these include montage details (right vs. left PFC stimulation; bipolar vs. high definition electrodes) and stimulation timing relative to the cognitive tasks (online vs. offline). For exercise, these factors include exercise mode (running vs. cycling), intensity (moderate vs. high), duration (length of the bout), and exercise timing relative to the cognitive tasks (during vs. immediately following vs. after a short delay of exercise). In the combined tDCS and exercise condition, it is possible that the order of exercise and stimulation relative to each other impacts the efficacy of these techniques on EF task performance. Here, we used a design where exercise preceded tDCS, which was presented while participants performed cognitive tasks. Alternatively, both tDCS could have been administered during the treadmill activity or simultaneous exercise and neurostimulation, or tDCS could have preceded the exercise activity [93]. Both alternatives are worthy of additional investigation to gain a deeper understanding of the interactive effects of each enhancement technique. Finally, it is worth noting that participants exercised at moderate intensity for 16 min total, even though many studies indicate that cognitive improvement comes about with a minimum of 20 min of moderate-intensity exercise. Future work might explore the interaction of tDCS and time on an exercise activity. We present just one set of parameters that may be implemented in a multimodal study; future work might example new combinations of each within the context of combined acute exercise and tDCS designs.

Third, it is possible that we might have observed more robust effects with more sensitive measurement tools (e.g., measuring brain activity). Indeed, previous work has shown changes in neural and physiological signatures, absent behavior effects [19]. Future efforts examining multiple cognitive enhancement techniques should consider including brain-based metrics to evaluate cognitive processes.

## 5. Conclusions

There is a rising interest among healthy individuals in neuroenhancement tools to benefit cognitive functions, like inhibition, working memory, and sustained attention. The present findings showed that inhibition was selectively impacted by acute aerobic exercise but not tDCS, whereas working memory was improved by both acute aerobic exercise and tDCS. Conversely, sustained attention was not impacted by acute aerobic exercise or tDCS. In addition, no additive effects of combining acute aerobic exercise and tDCS emerged under the current experimental conditions, suggesting that the influence each of these interventions has on executive function abilities may not be interactive or additive. It is possible that there are limits of performance for young healthy individuals on the cognitive tests used and that this decreased the likelihood of observing any additive or synergistic effects. We believe that understanding the unique and combined contributions of these techniques may have important implications for cognitive optimization among groups with executive dysfunction or under natural conditions where EF is compromised, such as under extreme stress or due to age-related decline.

## Figures and Tables

**Table 1:** Descriptive statistics of participants’ demographics, anthropometry, baseline cardiorespiratory fitness, and tDCS expectations and sensations.

**Demographics and Anthropometry (Session 1)**							
**Exercise**	**tDCS**	**Age (Years)**	**Sex (No. Females)**	**Height (cm)**	**Weight (kg)**		
Exercise	Active	22.58	12	169.2 ± 12.0	71.2 ± 21.0		
Exercise	Sham	22.21	18	165.2 ± 10.2	65.4 ± 18.8		
Seated	Active	23.54	12	170.4 ± 9.7	72.7 ± 27.5		
Seated	Sham	20.83	18	167.0 ± 10.3	66.1 ± 10.0		
**Cardiorespiratory Fitness Baseline (Session 1)**							
**Exercise**	**tDCS**	**VO_2_ Peak (mL/kg/min)**	**Baseline HR (bpm)**	**Max HR (bpm)**	**Grade (% incline)**	**Speed (mph)**	**Duration (s)**
Exercise	Active	50.3 ± 10.0	77.6 ± 13.3	185.3 ± 15.4	11 +/− 3.1	5.5 +/− 0.6	610.8 +/− 156.7
Exercise	Sham	46.6 ± 9.9	77.0 ± 17.1	186.8 ± 8.1	10.5 +/− 3.6	5.4 +/− 0.5	572.8 +/− 190
Seated	Active	49.8 ± 8.0	76.6 ± 9.3	189.9 ± 9.2	10.8 +/− 3.5	5.7 +/− 0.6	601.9 +/− 152.4
Seated	Sham	45.1 ± 10.5	81.3 ± 13.6	191.5 ± 9.8	11 +/− 1.8	5.6 +/− 0.5	609.1 +/− 80
**tDCS Expectations (Sessions 1 and 2)**							
**Exercise**	**tDCS**	**Before Session 1**	**After Session 2**				
		**(Out of 10)**	**(Out of 10)**				
Exercise	Active	4.50	5.33				
Exercise	Sham	4.29	4.25				
Seated	Active	4.71	5.57				
Seated	Sham	4.33	5.08				
**tDCS Sensations (Session 2)**							
**Exercise**	**tDCS**	**Distracting**	**Itchy**	**Pain**	**Heat**	**Discomfort**	
		**(Yes = 1/No = 0)**	**(Out of 10)**	**(Out of 10)**	**(Out of 10)**	**(Out of 10)**	
Exercise	Active	0.33	2.88	1.33	1.42	2.38	
Exercise	Sham	0.17	2.38	1.75	1.29	2.08	
Seated	Active	0.22	3.43	1.26	2.09	2.52	
Seated	Sham	0.21	2.79	1.83	1.71	2.38	

**Table 2:** Linear mixed-effect model output of Aerobic Exercise (Seated vs. Exercise), tDCS (Active vs. Sham), and Congruency (Incongruent vs. Congruent) on the flanker task for accuracy, median correct response time, correct response time standard deviation, and ex-Gaussian parameters (μ, σ, and τ). Bolded model parameters indicate significant findings (i.e., *t* > |2.00|). SE = Standard Error.

Predictor	Fixed Effects			Random Effects
	Coefficient	SE	t-Value	By-Subject Variance
**Accuracy**				
Intercept	−0.001	0.007	−0.161	0.003074
Aerobic Exercise	−0.015	0.013	−1.149	
tDCS	−0.014	0.013	−1.082	
Congruency	−0.003	0.006	−0.481	
Aerobic Exercise x tDCS	−0.011	0.026	−0.405	
Aerobic Exercise x Congruency	0.001	0.013	0.078	
tDCS x Congruency	−0.007	0.013	−0.503	
Aerobic Exercise x tDCS x Congruency	−0.014	0.026	−0.525	
**Correct Median Response Time (ms)**				
Intercept	4.208	2.773	1.518	690.07
Aerobic Exercise	−4.865	5.546	−0.877	
tDCS	−7.010	5.546	−1.264	
Congruency	−1.292	1.415	−0.913	
Aerobic Exercise x tDCS	0.625	11.091	0.056	
Aerobic Exercise x Congruency	−2.938	2.830	−1.038	
tDCS x Congruency	−0.938	2.830	−0.331	
Aerobic Exercise x tDCS x Congruency	0.500	5.659	0.088	
**Correct Response Time Standard Deviation (ms)**				
**Intercept**	**5.963**	**1.513**	**3.941**	**144.8**
**Aerobic Exercise**	**−6.420**	**3.026**	**−2.122**	
tDCS	−5.039	3.026	−1.665	
Congruency	−2.890	1.767	−1.635	
Aerobic Exercise x tDCS	3.711	6.052	0.613	
Aerobic Exercise x Congruency	−3.171	3.535	−0.897	
tDCS x Congruency	1.419	3.535	0.401	
Aerobic Exercise x tDCS x Congruency	−10.121	7.070	−1.431	
**ex-Gaussian μ**				
Intercept	2.267	2.524	0.898	484.4
Aerobic Exercise	−1.371	5.048	−0.272	
tDCS	−5.969	5.048	−1.183	
Congruency	−0.288	2.302	−0.125	
Aerobic Exercise x tDCS	−2.284	10.096	−0.226	
Aerobic Exercise x Congruency	0.315	4.603	0.068	
tDCS x Congruency	−1.864	4.603	−0.405	
Aerobic Exercise x tDCS x Congruency	1.132	9.207	0.123	
**ex-Gaussian σ**				
**Intercept**	**6.167**	**1.073**	**5.746**	**49.56**
Aerobic Exercise	−3.748	2.147	−1.746	
tDCS	−4.275	2.147	−1.991	
Congruency	−2.173	1.595	−1.362	
Aerobic Exercise x tDCS	−1.581	4.293	−0.368	
Aerobic Exercise x Congruency	2.482	3.189	0.778	
tDCS x Congruency	−0.829	3.189	−0.260	
Aerobic Exercise x tDCS x Congruency	−8.989	6.379	−1.409	
**ex-Gaussian τ**				
Intercept	1.556	1.782	0.873	165.5
Aerobic Exercise	−3.934	3.563	−1.104	
tDCS	−1.643	3.563	−0.461	
Congruency	−0.566	2.409	−0.235	
Aerobic Exercise x tDCS	3.420	7.127	0.480	
Aerobic Exercise x Congruency	−3.365	4.817	−0.699	
tDCS x Congruency	2.148	4.817	0.446	
Aerobic Exercise x tDCS x Congruency	−3.643	9.634	−0.378	

**Table 3:** Linear mixed-effect model output of Aerobic Exercise (Seated vs. Exercise), tDCS (Active vs. Sham), and Congruency (Incongruent vs. Congruent) on the n-back task for accuracy, A’, Grier’s β, median correct response time, correct response time standard deviation, and ex-Gaussian parameters (μ, σ, and τ). Bolded model parameters indicate significant findings (i.e., *t* > |2.00|). SE = Standard Error. Split across two panels is N-Level (2-Back vs. 4-Back, on the left and right, respectively). Error bars reflect standard errors of the mean.

Predictor	Fixed Effects			Random Effects	
	Coefficient	SE	t-Value	By-Subject Variance	By-Sequence Variance
**Average Accuracy**					
**Intercept**	**0.049**	**0.007**	**7.498**	**0.00160**	**0.00006**
Aerobic Exercise	0.021	0.011	1.877		
tDCS	0.012	0.011	1.111		
N-Level	0.009	0.007	1.189		
Aerobic Exercise x tDCS	−0.003	0.022	−0.114		
Aerobic Exercise x N-Level	−0.008	0.015	−0.515		
tDCS x N-Level	0.021	0.015	1.443		
Aerobic Exercise x tDCS x N-Level	−0.015	0.030	−0.522		
**Discriminability (A’)**					
**Intercept**	**0.040**	**0.009**	**4.452**	**0.00067**	
**Aerobic Exercise**	**0.041**	**0.018**	**2.266**		
tDCS	0.031	0.018	1.730		
N-Level	−0.015	0.017	−0.852		
Aerobic Exercise x tDCS	0.031	0.036	0.843		
Aerobic Exercise x N-Level	−0.032	0.035	−0.919		
tDCS x N-Level	0.008	0.035	0.237		
Aerobic Exercise x tDCS x N-Level	−0.091	0.070	−1.307		
**Response Bias (Grier’s β)**					
Intercept	0.023	0.038	0.612	0.007986	0.005785
Aerobic Exercise	0.023	0.032	0.729		
tDCS	−0.043	0.032	−1.355		
N-Level	−0.019	0.026	−0.703		
Aerobic Exercise x tDCS	0.097	0.064	1.510		
Aerobic Exercise x N-Level	−0.060	0.053	−1.135		
**tDCS x N-Level**	**−0.131**	**0.053**	**−2.493**		
Aerobic Exercise x tDCS x N-Level	0.063	0.105	0.593		
**Correct Median Response Time (ms)**					
**Intercept**	**80.55**	**13.46**	**5.99**	**11888.3**	**147.4**
Aerobic Exercise	−9.66	24.63	−0.39		
tDCS	−22.66	24.63	−0.92		
N-Level	−4.27	10.54	−0.40		
Aerobic Exercise x tDCS	−36.05	49.25	−0.73		
Aerobic Exercise x N-Level	−26.96	21.09	−1.28		
tDCS x N-Level	14.45	21.09	0.69		
Aerobic Exercise x tDCS x N-Level	−37.32	42.17	−0.89		
**Correct Response Time Standard Deviation**					
**Intercept**	**31.51**	**6.67**	**4.72**	**2071.88**	**58.28**
Aerobic Exercise	−9.73	11.47	−0.85		
tDCS	−5.05	11.47	−0.44		
N-Level	−12.60	6.72	−1.87		
Aerobic Exercise x tDCS	−24.24	22.94	−1.06		
Aerobic Exercise x N-Level	24.24	13.45	1.80		
tDCS x N-Level	−12.14	13.45	−0.90		
Aerobic Exercise x tDCS x N-Level	−28.36	26.90	−1.05		
**ex-Gaussian μ**					
**Intercept**	**57.25**	**12.18**	**4.70**	**10253**	
Aerobic Exercise	2.68	24.36	0.11		
tDCS	−15.49	24.36	−0.64		
N-Level	7.06	12.89	0.55		
Aerobic Exercise x tDCS	−25.64	48.72	−0.53		
Aerobic Exercise x N-Level	−33.97	25.78	−1.32		
tDCS x N-Level	10.71	25.78	0.42		
Aerobic Exercise x tDCS x N-Level	2.92	51.55	0.06		
**ex-Gaussian σ**					
**Intercept**	**20.22**	**5.32**	**3.80**	**1464**	
Aerobic Exercise	2.87	10.64	0.27		
tDCS	3.53	10.64	0.33		
N-Level	−4.08	7.23	−0.56		
Aerobic Exercise x tDCS	−11.42	21.29	−0.54		
Aerobic Exercise x N-Level	5.73	14.46	0.40		
tDCS x N-Level	−6.16	14.46	−0.43		
Aerobic Exercise x tDCS x N-Level	−36.64	28.92	−1.27		
**ex-Gaussian τ**					
**Intercept**	**24.83**	**6.42**	**3.86**	**1504**	
Aerobic Exercise	−1.25	12.85	−0.10		
tDCS	0.41	12.85	0.03		
N-Level	−13.00	10.12	−1.28		
Aerobic Exercise x tDCS	−3.42	25.70	−0.13		
Aerobic Exercise x N-Level	8.98	20.24	0.44		
tDCS x N-Level	−29.95	20.24	−1.48		
Aerobic Exercise x tDCS x N-Level	−27.36	40.48	−0.68		
